# Proptosis of the Left Broad Ligament as a Cause of Constipation

**Published:** 1911-07-22

**Authors:** 


### Gynæcology.

### PROPTOSIS OF THE LEFT BROAD LIGAMENT AS A CAUSE OF CONSTIPATION.

Although it has long been recognised that constipation is not disease in itself, but merely a symptom of some underlying condition, it is only recently that attempts have been made to elucidate the pathological anatomy of those defects in the motor mechanism of the bowel with which constipation is associated. The result has been that the surgeons have invaded yet another of the medical preserves, and with some success apparently.

### Some Anatomical Points.

A paper by Mr. Ernest Miles gives an interesting account of a number of cases of constipation in women, in all of which it was found to be the result of the pressure of a displaced and prolapsed broad ligament upon the rectum. Mr. Miles operated upon no fewer than 150 of these cases in the manner presently to be described, and in the vast majority was able completely to relieve their symptoms. And, moreover, the recovery so brought about has proved to be of permanent character, for some of the operations were carried out so long as eight years ago.

It will be of interest to describe the condition of the parts as revealed at the operations on these cases.

The exact position of the misplaced broad ligament is really one of backward rotation through one-quarter of a circle, accompanied by a slight dropping downwards in the pelvis. The free edge of the ligament, instead of occupying a vertical position (in the upright posture), looks directly backwards, and its anterior surface is directed upwards. The left half of the pelvis therefore becomes a closed space with, as it were, a lid formed by the left broad ligament. At the posterior part of this lid is a gap through which the sigmoid passes as best it can. With a large (*i.e.* distended) sigmoid and a taut broad ligament the onward passage of the fæces obviously becomes a matter of some difficulty, and constipation is the inevitable result. Granted that such a state of affairs may exist, it becomes apparent that the constant pressure against the sigmoid is likely to lead to inflammatory reaction, resulting in the formation of adhesions between the bowel and that part of the broad ligament in intimate contact with it. These adhesions may be close and widespread, or slight bands connecting with the appendices epiploicæ. In point of fact, all degrees of adhesion are found in different cases.

### The Position of the Colon.

Taking its share in the discomfort and disturbance will be the left ovary, which, forced into an unnatural position in the pelvis by the rotation and descent of the broad ligament and by the pressure of the now distended colon, becomes inflamed, tender, and adherent. In fact, there can be little doubt that here we have an explanation for those frequent cases of tender prolapsed ovary which so often call for surgical attention.

The condition of the parts fully explains the presence of various other symptoms which accompany the constipation. Pain is usually present, and as it results from the peritoneal adhesions it is most often referred to the left lower quadrant of the abdomen. At the menstrual periods the condition of the ovary is likely to give rise to discomfort owing to its increased vascularity at that time.

Following upon the chronic constipation there will be malaise, headaches, and the other symptoms of auto-toxæmia, which ultimately may become so severe as to cause the patient to lead a life of chronic invalidism.

### The Operative Treatment.

The operation suggested for dealing with these cases consists in opening the abdomen by a subumbilical incision, the patient being in the Trendelenberg position. The condition of the pelvic organs is then carefully investigated; delicate adhesions are separated by gentle sponge pressure; the denser ones must be carefully ligatured and cut away. The rectum having been freed in this way, the broad ligament is now fully exposed and divided at its outer part beyond the termination of the Fallopian tube. The incision is then found to stretch open and take a V-shape, the apex of the V being at the pelvic floor.

It will now be found that the ovary can readily be brought up into the wound. There may be one or more small cysts in it, which are either pricked or excised, or the ovary may be so damaged that its removal is deemed advisable. If so, its pedicle is transfixed, ligatured, and divided, and the stump buried between the two layers of the broad ligament which are then sewn together over it. The suture is then carried along the edge of the V and the peritoneal edges brought into close apposition. The broad ligament will now be almost completely obliterated, and the rectum will have an unimpeded path through the pelvis.

### Results of Operation.

No bad result has been found to follow the removal of whatever support the uterus may receive from the ligament, and once the bowel has got accustomed to the new condition of affairs and the habit of regular daily evacuation established, the patients show a remarkable improvement.

Whether other surgeons will find the cases suitable for operation easy to recognise and as easy to deal with remains to be seen. In any case, the operative treatment of chronic constipation is a subject which has now attained a considerable importance, and Mr. Miles' paper must be carefully studied by all those interested in this particular branch of surgery, more especially if they hold the view that ablation of the whole of the large intestine is a suitable manner in which to deal with such cases.

